# Reduction of the Risk of Hepatocellular Carcinoma over Time Using Direct-Acting Antivirals: A Propensity Score Analysis of a Real-Life Cohort (PITER HCV)

**DOI:** 10.3390/v16050682

**Published:** 2024-04-26

**Authors:** Maria Giovanna Quaranta, Luisa Cavalletto, Francesco Paolo Russo, Vincenza Calvaruso, Luigina Ferrigno, Alberto Zanetto, Benedetta Mattioli, Roberta D’Ambrosio, Valentina Panetta, Giuseppina Brancaccio, Giovanni Raimondo, Maurizia Rossana Brunetto, Anna Linda Zignego, Carmine Coppola, Andrea Iannone, Elisa Biliotti, Elena Rosselli Del Turco, Marco Massari, Anna Licata, Francesco Barbaro, Marcello Persico, Filomena Morisco, Maurizio Pompili, Federica Cerini, Massimo Puoti, Teresa Santantonio, Antonio Craxì, Loreta A. Kondili, Liliana Chemello, on behalf of PITER Collaborating Investigators

**Affiliations:** 1 Center for Global Health, Istituto Superiore di Sanità (ISS), 00161 Rome, Italy; mariagiovanna.quaranta@iss.it (M.G.Q.); luigina.ferrigno@iss.it (L.F.); benedetta.mattioli@iss.it (B.M.); 2 Department of Medicine-DIMED, Clinica Medica 5, Refering Regional Center for Liver Diseases, University Hospital, Padua University, 35122 Padova, Italy; luisa.cavalletto@unipd.it; 3 Department of Surgery, Oncology and Gastroenterology, Gastroenterology Unit, University of Padua, 35122 Padua, Italy; francescopaolo.russo@unipd.it (F.P.R.); alberto.zanetto@unipd.it (A.Z.); 4 Gastroenterology and Hepatology Unit, PROMISE, University of Palermo, 90133 Palermo, Italy; vincenza.calvaruso@unipa.it (V.C.); antonio.craxi@unipa.it (A.C.); 5 Division of Gastroenterology and Hepatology, Foundation IRCCS Ca’ Granda Ospedale Maggiore Policlinico, 20122 Milan, Italy; roberta.dambrosio@policlinico.mi.it; 6 L’altrastatistica S.r.l., Consultancy & Training, Biostatistics Office, 00174 Rome, Italy; valentina.panetta@laltrastatistica.com; 7 Infectious Diseases Unit, Department of Molecular Medicine, University of Padua,35122 Padua, Italy; ggbrancaccio@gmail.com; 8 Department of Internal Medicine, University Hospital of Messina, 98122 Messina, Italy; raimondo@unime.it; 9 Department of Clinical and Experimental Medicine, University Hospital of Pisa, 56126 Pisa, Italy; brunettomaurizia@gmail.com; 10 Department of Experimental and Clinical Medicine, Interdepartmental Centre MASVE, University of Florence, 50121 Florence, Italy; annalinda.zignego@unifi.it; 11 Department of Hepatology, Gragnano Hospital, 80054 Naples, Italy; carminecoppola@alice.it; 12 Section of Gastroenterology, Department of Emergency and Organ Transplantation, University of Bari, 70121 Bari, Italy; ianan@hotmail.it; 13 Infectious and Tropical Medicine Unit, Department of Public Health and Infectious Diseases, “Policlinico Umberto I” Hospital, Sapienza University of Rome, 00161 Rome, Italy; elisabiliotti@yahoo.it; 14 Infectious Diseases Unit, Department for Integrated Infectious Risk Management, IRCCS Azienda Ospedaliero-Universitaria di Bologna, 40138 Bologna, Italy; erossellidelturco@gmail.com; 15 Malattie Infettive, Azienda Unità Sanitaria Locale, IRCCS di Reggio Emilia, 42123 Reggio Emilia, Italy; massari.marco@ausl.re.it; 16 Infectious Diseases Unit, DIBIMIS, University of Palermo, 90133 Palermo, Italy; anna.licata@unipa.it; 17 Department of Medicine, Infectious Diseases Unit, University of Padua, 35122 Padua, Italy; francesco.barbaro@aopd.veneto.it; 18 Internal Medicine and Hepatology Division, Department of Medicine, Surgery and Dentistry, “Scuola Medica Salernitana”, University of Salerno, 84084 Baronissi, Italy; mpersico@unisa.it; 19 Gastroenterology Unit, Federico II University, 80138 Naples, Italy; filomena.morisco@unina.it; 20 Internal Medicine and Gastroenterology, Fondazione Policlinico Universitario Agostino Gemelli IRCCS, Largo Agostino Gemelli, 00136 Rome, Italy; maurizio.pompili@unicatt.it; 21 Department of Clinical Sciences and Community Health, University of Milan, Hepatology Unit, San Giuseppe Hospital, 20123 Milan, Italy; federica.cerini@unimi.it; 22 Infectious Disease Unit, Niguarda Hospital, 20142 Milan, Italy; massimopuoti@libero.it; 23 School of Medicine, University of Milano-Bicocca, 20126 Milan, Italy; 24 Infectious Diseases Unit, Department of Clinical and Surgical Sciences, University of Foggia, AOU Policlinico Riuniti Foggia, 71122 Foggia, Italy; t.santantonio@unifg.it; 25 Internal Medicine, UniCamillus-Saint Camillus International University of Health Sciences, 00131 Rome, Italy

**Keywords:** hepatocellular carcinoma, hepatitis C virus, direct-acting antiviral, sustained virological response, real-life cohort

## Abstract

The treatment of hepatitis C virus (HCV) with direct-acting antivirals (DAA) leads to high sustained virological response (SVR) rates, but hepatocellular carcinoma (HCC) risk persists in people with advanced liver disease even after SVR. We weighted the HCC risk in people with cirrhosis achieving HCV eradication through DAA treatment and compared it with untreated participants in the multicenter prospective Italian Platform for the Study of Viral Hepatitis Therapies (PITER) cohort. Propensity matching with inverse probability weighting was used to compare DAA-treated and untreated HCV-infected participants with liver cirrhosis. Kaplan–Meier analysis and competing risk regression analysis were performed. Within the first 36 months, 30 de novo HCC cases occurred in the untreated group (*n* = 307), with a weighted incidence rate of 0.34% (95%CI: 0.23–0.52%), compared to 63 cases among SVR patients (*n* = 1111), with an incidence rate of 0.20% (95%CI: 0.16–0.26%). The 12-, 24-, and 36-month HCC weighted cumulative incidence rates were 6.7%, 8.4%, and 10.0% in untreated cases and 2.3%, 4.5%, and 7.0% in the SVR group. Considering death or liver transplantation as competing events, the untreated group showed a 64% higher risk of HCC incidence compared to SVR patients (SubHR 1.64, 95%CI: 1.02–2.62). Other variables independently associated with the HCC occurrence were male sex, increasing age, current alcohol use, HCV genotype 3, platelet count ≤ 120,000/µL, and albumin ≤ 3.5 g/dL. In real-life practice, the high efficacy of DAA in achieving SVR is translated into high effectiveness in reducing the HCC incidence risk.

## 1. Introduction

Chronic hepatitis C virus (HCV) infection is a global public health challenge as it is a primary cause of liver disease-related morbidity and mortality. Worldwide, around 50 million people live with HCV, with about 1.0 million new infections annually [1]. WHO estimated that in 2022, around 242,000 people died from hepatitis C, primarily due to cirrhosis and hepatocellular carcinoma (HCC) [2]. The achievement of sustained virologic response (SVR), characterized by undetectably low HCV RNA levels, has been associated with a reduction in liver-related complications, an improvement in health-related quality of life, and a reduced need for liver transplantation in individuals with chronic hepatitis C [3]. Direct-acting antivirals (DAA) represent a class of drugs specifically designed to inhibit viral replication by targeting key viral proteins involved in the viral life cycle [4]. DAA drastically changed the treatment efficacy of chronic HCV-related disease, reaching a high sustained virological response (SVR) rate of over 98% [5,6]. These treatments not only improve liver fibrosis and function but also reduce portal hypertension in most cases [7,8,9,10], have an excellent safety profile, and are generally well tolerated [5,6]. Real-life data have demonstrated similar or even higher rates of DAA efficacy to induce HCV clearance and the improvement of liver-related disease [10,11]. However, efficacy is not the same as effectiveness, and the risk of complications in cases with advanced liver disease was not avoided. Several observational studies conducted by different research groups, based on medium- and long-term prospective evaluations [12,13], have suggested that DAA-induced SVR is associated with a reduced risk of HCC incidence, even if people with moderate or severe stages of liver disease remain at risk for HCC and must continue surveillance after viral eradication [10,14,15,16,17,18,19,20].

Earlier reports indicated an unexpectedly high rate of HCC occurrence in HCV-positive individuals who achieved SVR following DAA therapy [21,22]. In these studies, the authors hypothesized that the rapid HCV reduction could potentially weaken the immune surveillance system for tumors, leading to HCC development. However, it must be considered that HCC occurrence after DAA therapy may be linked to treating patients at intrinsically higher cancer risk, unlike IFN treatment, which was attempted only in patients with well-compensated cirrhosis, often without clinically significant portal hypertension, and with lower HCC risk. Indeed, prospective studies comparing lower HCC incidences in IFN- vs. DAA-treated participants largely hinge on differences in group populations, as DAA allows treatment in individuals with more advanced liver diseases and/or comorbidities who were previously contraindicated to IFN-based therapy. 

Given the ethical and practical constraints of conducting a DAA versus placebo trial, the incidence rate of HCC in DAA-treated participants over time post-HCV eradication in various cohorts, alongside the reduction in HCV mortality rates following DAA use, serve as indirect evidence supporting the decreasing risk of HCC after viral eradication trough DAA treatment [23,24]. 

In our previous study, conducted in the Italian Platform for the Study of Viral Hepatitis Therapies (PITER) cohort, we identified older age, genotype HCV-3, the presence of diabetes, and pre-treatment low platelet count and albumin level as factors independently associated with HCC occurrence after SVR, with the best predictability given by the association of platelet count and albumin level, analyzed prospectively at long-term follow-up (FU) after SVR [14]. In the present study, we took advantage of the data prospectively collected in the PITER cohort to evaluate the real-life effectiveness of DAA in reducing the risk of HCC development through a propensity-matched analysis of DAA-treated and untreated HCV-infected participants with liver cirrhosis over a three-year follow-up period.

## 2. Methods

### 2.1. Study Population

The study population included participants with chronic HCV infection who were consecutively enrolled in the ongoing, prospective PITER cohort from 60 tertiary centers in liver and infectious diseases across university or general hospitals throughout Italy. For each participant, demographic, clinical, and laboratory data were recorded in a dedicated electronic Case Report Form during the participant’s enrolment visit and throughout the FU period, in accordance with the real-life practice of each clinical center. The time of baseline data collection was the enrolment visit for the untreated participants and the pre-treatment visit for the treated group. Data quality was ensured through periodic remote monitoring, which included the resolution of specific queries. For the purpose of this study, the database was frozen as of April 2023.

### 2.2. Inclusion and Exclusion Criteria

We assessed participants enrolled in the PITER cohort between June 2014 and April 2023 who had a confirmed diagnosis of liver cirrhosis and were either treated with DAA therapy or left untreated, for inclusion in this study. The control group comprised participants who were enrolled but not treated with DAAs during the initial three years of DAA availability, despite their progressive liver disease and was utilized to assess the risk of HCC. All untreated participants included in the analysis did not receive DAA therapy during the 36-month follow-up period. Liver cirrhosis was defined by either liver biopsy (Metavir ≥ 4 or Ishak score ≥ 6) or Transient Elastography (liver stiffness measurement [LSM] > 12.5 kPa) [25] or biochemical and/or imaging indicative of clinically significant portal hypertension (i.e., presence of esophageal and/or gastric varices and/or platelet count ≤ 150,000/µL with splenomegaly). People classified as Child–Pugh–Turcotte (CPT) class A or B with compensated liver disease or those with a history of decompensation but showing a stable clinical condition with compensation within the preceding 12-month period were included. Exclusion criteria comprised participants with a prior diagnosis of HCC or pre-enrolment unclassified nodular lesions, individuals who had undergone liver transplantation (LT) or were on an LT waiting list, as well as those with HIV or HBV coinfection. Additionally, people who were treated but did not achieve an SVR were also excluded from the analysis.

### 2.3. Hepatocellular Carcinoma Occurrence

HCC surveillance was performed on all participants by ultrasound examination every six months. In addition, HCC was diagnosed by needle biopsy or by non-invasive criteria according to international guidelines [26]. For untreated participants, incident cases of HCC diagnosed for the first time after the enrolment date were identified. In the SVR group, HCC cases occurring after the end of treatment (EOT) were considered to be de novo HCC.

### 2.4. Statistical Analysis 

To address the imbalance between the untreated and the SVR groups, a propensity score (PS) was calculated using a probit regression model including the following covariates: age (years), bilirubin (mg/dL), platelets count (cells/µL), albumin level (g/dL), INR, creatinine (mg/dL), male sex, BMI, alcohol use, previous decompensation, and genotype HCV-3.

The standardized mean difference (Std-Diff) was calculated to ensure the balance after weighting. Inverse probability weighting (IPW) was applied to weight comparisons. The Std-Diff calculations in unweighted and weighted samples ensured a balance of baseline covariates between the two groups, with a Std-Diff < 0.1 considered indicative of reliable balancing.

The EOT was considered as the starting time point for SVR participants, while the enrollment date was used for untreated participants. A Fine–Gray competing-risks regression model was utilized to evaluate the effect of demographic data and clinical variables on the risk of HCC occurrence, considering death or LT as competing events (i.e., events that could prevent the event of interest). Both univariable and multivariable analyses were conducted. In the multivariable analysis, a forward stepwise selection method was used to obtain robust results. Results were expressed as a sub-hazard ratio (subHR) with a corresponding 95% confidence interval (95%CI). 

For all analyses, a *p* < 0.05 was considered statistically significant. All statistical analyses were performed using STATA version 16.1 (Stata Corp., College Station, TX, USA).

## 3. Results

### 3.1. Comparison of HCC Incidence rate between Untreated and SVR Participants

Data from 307 untreated participants and 1111 DAA-treated participants who achieved the SVR were included. As shown in Table 1, all the variables analyzed were well balanced between the two groups after applying inverse probability weighting (IPW) (Weighted Std-Diff < 0.1). Thanks to the propensity score, the untreated and DAA-treated groups were match-balanced in terms of liver disease severity (CPT class A: 88.8% vs. 87.1%; CTP class B: 11.2% vs. 12.9%, respectively). 

**Figure 1:**
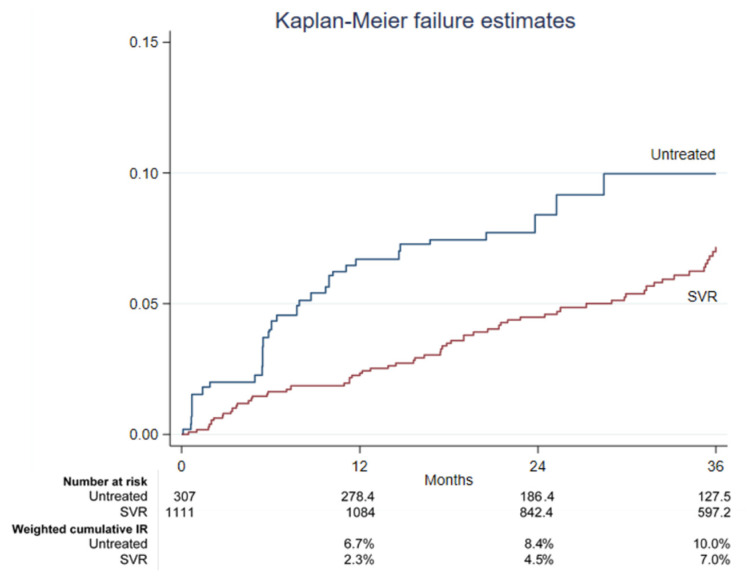
Kaplan–Meier failure estimates in untreated and SVR groups.

In the first 36 months (starting from the EOT for the SVR participants and from the enrollment date for untreated cases), there were 30 de novo HCC diagnoses in the untreated group, with a weighted incidence rate of 0.34% (95%CI: 0.23–0.52%), and 63 in the SVR group, with a weighted incidence rate of 0.20% (95%CI: 0.16–0.26%). The overall HCC incidence rate is reported in Figure 1. The weighted cumulative incidence rates at 12, 24, and 36 months were 6.7%, 8.4%, and 10.0% respectively, in the untreated group, and 2.3%, 4.5%, and 7.0%, respectively, in the SVR group.

### 3.2. Impact of Effective DAA Therapy on the Risk of HCC Incidence

Considering death or liver transplantation as competing events, untreated participants showed a 64% higher risk of developing HCC compared to SVR participants (SubHR 1.64, 95%CI 1.02–2.62) (Table 2). Thanks to the propensity score, the treatment considered in the univariable model accounts for possible confounding factors. A multivariable model was applied to underline the effect of other variables adjusted for the treatment. After adjusting for the confounding effect of each variable identified through stepwise forward selection, other variables independently associated with the HCC occurrence were male sex (SubHR 1.94, 95%CI: 1.23–3.06), increasing age (SubHR 1.03, 95%CI: 1.01–1.05), current alcohol use (SubHR 2.3, 95%CI: 1.18–4.49), genotype HCV-3 (SubHR 2.27, 95%CI: 1.09–4.70), platelet count ≤ 120,000/µL (SubHR 1.71, 95%CI: 1.07–2.73) and albumin level ≤ 3.5 g/dL (SubHR 1.95, 95%CI: 1.23–3.09) (Table 2).

## 4. Discussion

The data from this study, based on a prospectively evaluated propensity-matched analysis of a multicenter nationwide real-life clinical practice of 60 centers distributed throughout Italy, clearly demonstrate that HCV clearance significantly reduces the risk of HCC incidence compared to untreated HCV-positive individuals. Before DAA use in 2014, Italy had the highest burden of HCV liver-related complications and deaths [27]. These data were also confirmed by the greater number of HCV-positive individuals treated with DAA in the 2015–2016 period in Europe, with a priority initially given to HCV-positive people with liver cirrhosis [28], with an overall SVR rate in real-life practice of over 96% [29], which is similar to the rates reported by clinical trials [5,6].

In our previous paper, we evaluated the HCC risk profile based on predictors monitored after HCV eradication by DAA in people with cirrhosis. We showed that the best predictor of HCC incidence after SVR was given by the association of two easy and accurate markers, undoubtedly associated with portal hypertension and liver function: platelet count and albumin level, analyzed prospectively at long-term FU after SVR [14]. In the present study, we focused on the risk of HCC development in participants with cirrhosis that eradicated HCV infection by DAA treatment compared to a group of untreated participants. The PITER cohort of consecutively enrolled individuals in care but not under treatment at the time of enrollment offers a database of prospectively collected cases with chronic hepatitis C and enables the comparison between treated and untreated participants during a 36-month FU for the estimation of HCC risk. Thus, the PITER cohort provides a unique opportunity to include a matched control group of untreated participants with HCV-related cirrhosis, since not all cases included at the beginning of the DAA era could be treated immediately after enrolment due to the limited availability of DAA during the 2014–2016 period in Italy.

In this analysis, we observed weighted cumulative incidence rates of HCC after 12, 24, and 36 months of 2.3%, 4.5%, and 7.0% in the DAA-treated participants who achieved SVR and 6.7%, 8.4%, and 10.0% in untreated cases. These data were very similar to those obtained from the prospective cohort of the HCV Sicily network (RESIST-HCV), which showed an HCC incidence of 2.1% and 7.8% in people with CPT class A and B, respectively, among SVR, but in 6.6% and 12.4% in the non-SVR group, respectively [19].

Consistent with this observation, several studies have shown that the risk for HCC incidence is significantly higher among people who did not achieve SVR compared with those who did [15,16]. Moreover, a protective role of SVR after DAA has been reported, compared to that obtained with IFN [12,13]. These observations also confirm the avoidance of de novo HCC onset after DAA, particularly in the CPT class A and SVR group of participants. However, although HCV eradication has promoted the reduction of HCC cases, its occurrence has not been eliminated, especially in people with severe liver fibrosis, highlighting the importance of initiating DAA treatment before the development of advanced cirrhosis [30,31]. Indeed, the evidence of older age, genotype 3, the presence of diabetes, low albumin level and platelet count, and relapse of viremia after DAA therapy remain important parameters independently associated with higher HCC incidence risk [14,19]. 

The greater number of HCC cases rose after DAA therapy in people with liver cirrhosis, which is related to the more advanced stage of liver disease and is a known risk factor, and this fact may explain the lower rates of HCC after IFN treatment, which was typically only administered to people with chronic hepatitis or well-compensated cirrhosis, mostly without clinically significant portal hypertension and thus with a lower HCC risk [13,32]. 

The treated population has changed significantly in the era of DAA and now includes many patients with other risk factors of HCC. Due to universal access to treatment regardless of liver disease stage, in addition to the clinical variables reflecting disease severity, it would be interesting to prospectively evaluate the role of social determinants of health as risk factors for the development and different clinical outcomes of HCC [33]. Further analysis focusing on this topic in the DAA-treated cohort is ongoing. 

Original observations reported in our study include the comparative evaluation of HCC risk after a balanced match between DAA-treated and untreated participants by propensity analysis according to demographic and virological data (age, sex, genotype HCV-3), as well as clinical variables serving as surrogate markers of liver function (bilirubin, platelet count, albumin, INR, creatinine and a history of previous decompensation), which are used in order to evaluate the effect of viral eradication and liver disease stage on the risk of HCC occurrence. The results of this comparative analysis revealed that viral elimination through DAA was associated with a 64% reduction in the risk of HCC development compared to untreated controls. Additionally, considering death or liver transplantation as competing events, factors independently associated with HCC incidence included male sex, increasing age, current alcohol use, genotype HCV-3, platelet count ≤ 120,000/µL, and albumin level ≤ 3.5 g/dL. 

These findings, comparing participants successfully treated with DAA or untreated participants over nearly three years of follow-up, strongly suggest the important role of achieving SVR through DAA therapy in reducing the risk of HCC in people with advanced liver disease and cirrhosis. However, the HCC risk is still considerable after SVR among individuals with cirrhosis, emphasizing the importance of closely monitoring the high-risk group of people with cirrhosis.

### Strength and Limitations

The major strengths of our study are the large cohort of participants, the consecutive enrolment, and the complete clinical characterization with specific essential real-life data obtained in the tertiary liver unit. 

The diffusion of DAA in Italy from the end of 2014 to 2016 was prioritized for people with F3 and F4 liver cirrhosis, with the majority represented by IFN-experienced cases and/or advanced liver disease. However, the magnitude of this class of people exceeded the offer of therapy in Italy, since not all people with F4 fibrosis stage could be treated at the start of DAA marketing in all clinical centers distributed in Italy. Thus, a group of participants enrolled in the PITER platform did not receive the DAA therapy during the first period of DAA distribution, despite their progressive liver disease. This allowed us to prospectively compare treated vs. untreated participants in the first three years of DAA administration. The untreated group was prospectively followed based on the same criteria used for the DAA-treated group and was match-balanced with the approach of inverse probability weighting. Therefore, we argue that our strategy minimizes bias due to different demographics and/or the severity of liver disease between the groups. 

However, in this study, we included only those individuals who achieved the SVR. We could not analyze the data of people who did not achieve HCV eradication since most of these individuals underwent a new antiviral treatment shortly after failing the first one. Additionally, due to the DAA response rate surpassing 96% after a second treatment course, it was not feasible to compare SVR-positive and SVR-negative participants. Moreover, even if the PITER cohort includes participants with a medium- to long-term FU period of up to 8.4 years, the comparison between DAA-treated and untreated participants is limited to a maximum FU period of 3 years. After this period, the number of untreated participants decreases significantly, making a comparative analysis unfeasible.

## 5. Conclusions

In conclusion, using the consecutive and multicenter enrolment of the PITER cohort, this study demonstrates the significant reduction of HCC incidence risk in people successfully treated with DAA with cirrhosis compared to untreated individuals over nearly three years of follow-up. These findings reinforced the importance of curing HCV in all people, particularly in individuals with advanced chronic liver disease in whom a virologic cure will reduce but not eliminate the HCC risk.

## Figures and Tables

**Table 1:** Descriptive values of variables included in the propensity model calculation.

		SVR (*n* = 1111)		Untreated (*n* = 307)			
		Mean	SD	Mean	SD	Unweighted Std-Diff	Weighted Std-Diff
Age (years)		63.7	11.73	67.16	10.28	−0.41	0.07
Bilirubin (mg/dL)		1.02	0.67	1.33	1.84	−0.23	−0.01
Platelet count (µL)		126,114.5	61,318.84	118,215.66	71,624.02	0.19	−0.05
Albumin level (g/dL)		3.88	0.52	3.77	0.6	0.24	−0.04
INR		1.1	0.23	1.18	0.37	−0.27	0.01
Creatinine (mg/dL)		0.81	0.21	0.94	0.74	−0.24	−0.04
		**N**	**%**	**N**	**%**	**Unweighted** **Std-Diff**	**Weighted** **Std-Diff**
Sex male		581	52.0	154	50.0	0.04	−0.02
BMI:	Underweight	11	1.0	3	1.0	0.001	0.03
	Normal weight	453	40.8	148	48.2	−0.15	−0.02
	Overweight	473	42.6	114	37.1	0.11	0.04
	Obese	174	15.7	42	13.7	0.06	−0.03
Alcohol use:	Never	797	71.7	193	62.9	0.19	−0.03
	Current	99	8.9	39	12.7	−0.12	0.0003
	Past	215	19.3	75	24.4	−0.12	0.03
HCV-genotype 3		71	6.4	20	6.5	−0.01	−0.003
Previous decompensation		113	10.2	67	21.8	−0.32	0.01

All the analyzed variables were well matched between the two groups after inverse probability weighting (IPW) (Weighted Std-Diff < 0.1). Body mass index (BMI) was categorized as underweight (<18.5), normal (≥18.5 and <25.0 kg/m^2^), overweight (≥25.0 and <30.0 kg/m^2^) and obese (≥30.0 kg/m^2^). SVR: sustained virological response; Std-Diff: standardized mean difference; INR: international normalized ratio; HCV: hepatitis C virus.

**Table 2:** Univariable and multivariable competing risk model results, outcome HCC.

		Univariable Analysis			Multivariable Analysis		
Variable		SubHR	95% CI	*p*-Level	SubHR	95% CI	*p*-Level
Untreated (ref. SVR)		1.64	1.02–2.62	**0.042**	1.56	0.97–2.54	0.067
Male sex (ref. female)		1.72	1.11–2.66	**0.014**	1.94	1.23–3.06	**0.004**
Age (increasing years)		1.03	1.01–1.05	**0.002**	1.05	1.02–1.07	**<0.001**
BMI overweight/obese		0.92	0.60–1.41	**0.714**	not in the final model		
(ref. under-normal weight)							
Alcohol use	Current	2.02	1.09–3.74	**0.026**	2.30	1.18–4.49	**0.015**
(ref. never)	Past	1.25	0.74–2.11	**0.398**	1.07	0.62–1.83	0.804
Genotype HCV-3 (ref. others)		1.74	0.89–3.40	**0.104**	2.27	1.09–4.70	**0.028**
IFN-experienced (ref. naive)		1.17	0.77–1.79	**0.462**	not in the final model		
Platelet count (ref. > 120,000/µL)		1.98	1.25–3.14	**0.004**	1.71	1.07–2.73	**0.024**
LSM (ref. < 20 kPa)		1.29	0.75–2.22	**0.350**	not in the final model		
Past decompensation (ref. no)		1.76	1.01–3.05	**0.045**	not in the final model		
Diabetes (ref. no)		1.30	0.82–2.07	**0.269**	not in the final model		
Albumin level (ref. > 3.5 g/dL)		2.34	1.50–3.65	**<0.001**	1.95	1.23–3.09	**0.004**

Body mass index (BMI) was categorized as underweight (<18.5), normal (≥18.5 and <25.0 kg/m^2^), overweight (≥25.0 and <30.0 kg/m^2^) and obese (≥30.0 kg/m^2^). SubHR: sub-hazard ratio; CI: confidence interval; SVR: sustained virological response; HCV: hepatitis C virus; LSM: liver stiffness measurement. Bold numbers indicated statistically significant values.

## Data Availability

By protocol, the property of the data is of participating clinical centers while Istituto Superiore di Sanità acts as a coordinating center for data management and analysis. Cumulative data are reported within the paper, whereas each patient’s data are not fully available and without restrictions for ethical reasons. LA Kondili (loreta.kondili@iss.it) is in charge of data management and the readers may contact her for specific data requests. She will provide the necessary ethical clearances for access to data. Interested readers may contact Carlo Petrini (carlo.petrini@iss.it) from the Ethical Committee of Istituto Superiore di Sanità to request access to patient-level data.

## Appendix A

### PITER Collaborating Investigators

Alessio AGHEMO (Department of Gastroenterology, IRCCS Humanitas Research Hospital IRCCS, Rozzano, Italy)
Pietro ANDREONE (Department of Internal Medicine, University of Modena and Reggio Emilia, Modena, Italy)
Leonardo BAIOCCHI (Hepatology Unit, University of Tor Vergata, Rome, Italy)
Paolo BONFANTI (School of Medicine and Surgery, University of Milano-Bicocca, Monza, Italy).
Pierluigi BLANC (Infectious Disease Unit, Santa Maria Annunziata Hospital, Florence, Italy)
Alessia CIANCIO (Gastroenterology Unit, Città della Salute e della Scienza of Turin, University Hospital, Turin, Italy)
Luchino CHESSA (Liver Unit, University Hospital, Monserrato, Cagliari, Italy)
Pisa, Italy)
Barbara COCO (Department of Clinical and Experimental Medicine, University Hospital of Pisa,
Roberta COPPOLA (Department of Hepatology, Gragnano Hospital, Gragnano (NA), Italy)
Valentina COSSIGA (Gastroenterology Unit, Federico II University, Naples, Italy)
Silvia CRETELLA (Infectious Diseases Unit, Department for Integrated Infectious Risk Management, IRCCS Azienda Ospedaliero-Universitaria di Bologna, Italy)
Fernando DE ANGELIS (Department of Internal Medicine, “Policlinico Umberto I” Hospital, Sapienza University of Rome, Rome, Italy)
Adriano DE SANTIS (Department of Internal Medicine, “Policlinico Umberto I” Hospital, Sapienza University of Rome, Rome, Italy)
Martina DE SIENA (Internal Medicine and Gastroenterology, Fondazione Policlinico Universitario Alessandro FEDERICO (Department of Hepato-Gastroenterology, University of Campania Luigi Vanvitelli, Naples, Italy)
Roberto FILOMIA (Department of Internal Medicine, University Hospital of Messina, Messina, Italy)
Giovanni Battista GAETA (Infectious Diseases, University of Campania “Luigi Vanvitelli”, Naples, Italy)
Ivan GENTILE (Department of Clinical Medicine and Surgery, University of Naples Federico II, Napoli, Italy)
Donatella IELUZZI (Liver Unit, University Hospital of Verona, Verona, Italy)
Pietro INVERNIZZI (Division of Gastroenterology and Center for Autoimmune Liver Diseases, San Gerardo Hospital, Monza, Italy)
Diletta LACCABUE (Department of Medicine and Surgery, University of Parma, Unit of Infectious Diseases and Hepatology, Azienda Ospedaliero-Universitaria di Parma, Italy)
Pietro LAMPERTICO (Division of Gastroenterology and Hepatology, Foundation IRCCS Ca’ Granda Ospedale Maggiore Policlinico, Milan, Italy)
Salvatore MADONIA (Department of Internal Medicine, Villa Sofia-Cervello Hospital, Palermo, Italy)
Mario MASARONE (Internal Medicine and Hepatology Unit, University of Salerno, Salerno)
Agostino Gemelli IRCCS, Rome, Italy)
Monica MONTI (Department of Experimental and Clinical Medicine, Interdepartmental Centre MASVE, University of Florence, Florence, Italy)
Giulia MORSICA (Department of Infectious Diseases, San Raffaele Hospital, Milan Italy)
Giustino PARRUTI (Infectious Diseases Unit, Spirito Santo General Hospital, Pescara, Italy)
Maria Grazia RUMI (Hepatology Unit, San Giuseppe Hospital, Milan, Italy)
Ilaria SERIO (Division of Internal Medicine, Hepatobiliary and Immunoallergic Diseases, IRCCS Azienda Ospedaliero-Universitaria di Bologna, Italy)
Ilaria SIMONELLI (L’altrastatistica srl, Consultancy & Training, Biostatistics Office, Rome, Italy)
Matteo TONNINI (Department of Medical and Surgical Sciences, University of Bologna, Bologna, Italy)
Xhimi TATA (Center for Global Health, Istituto Superiore Di Sanità, Rome, Italy)
Carlo TORTI (Department of Medical and Surgical Sciences, Infectious and Tropical Diseases Unit, “Magna Graecia” University, Catanzaro, Italy)

## References

[1] World Health Organization (2017). Global Hepatitis Report, 2017.

[2] (2024). World Health Organization Hepatitis C Fact Sheet. WHO Website (Online). https://www.who.int/news-room/fact-sheets/detail/hepatitis-c.

[3] Nahon P., Bourcier V., Layese R., Audureau E., Cagnot C., Marcellin P., Guyader D., Fontaine H., Larrey D., De Lédinghen V. (2017). Eradication of Hepatitis C Virus Infection in Patients with Cirrhosis Reduces Risk of Liver and Non-Liver Complications. Gastroenterology.

[4] Bhattacharjee C., Singh M., Das D., Chaudhuri S., Mukhopadhyay A. (2021). Current Therapeutics against HCV. Virusdisease.

[5] Afdhal N., Reddy K.R., Nelson D.R., Lawitz E., Gordon S.C., Schiff E., Nahass R., Ghalib R., Gitlin N., Herring R. (2014). Ledipasvir and Sofosbuvir for Previously Treated HCV Genotype 1 Infection. N. Engl. J. Med..

[6] Feld J.J., Jacobson I.M., Hézode C., Asselah T., Ruane P.J., Gruener N., Abergel A., Mangia A., Lai C.-L., Chan H.L.Y. (2015). Sofosbuvir and Velpatasvir for HCV Genotype 1, 2, 4, 5, and 6 Infection. N. Engl. J. Med..

[7] Afdhal N., Everson G.T., Calleja J.L., McCaughan G.W., Bosch J., Brainard D.M., McHutchison J.G., De-Oertel S., An D., Charlton M. (2017). Effect of Viral Suppression on Hepatic Venous Pressure Gradient in Hepatitis C with Cirrhosis and Portal Hypertension. J. Viral Hepat..

[8] Lens S., Baiges A., Alvarado-Tapias E., LLop E., Martinez J., Fortea J.I., Ibáñez-Samaniego L., Mariño Z., Rodríguez-Tajes S., Gallego A. (2020). Clinical Outcome and Hemodynamic Changes Following HCV Eradication with Oral Antiviral Therapy in Patients with Clinically Significant Portal Hypertension. J. Hepatol..

[9] Mandorfer M., Kozbial K., Schwabl P., Chromy D., Semmler G., Stättermayer A.F., Pinter M., Hernández-Gea V., Fritzer-Szekeres M., Steindl-Munda P. (2020). Changes in Hepatic Venous Pressure Gradient Predict Hepatic Decompensation in Patients Who Achieved Sustained Virologic Response to Interferon-Free Therapy. Hepatology.

[10] Carrat F., Fontaine H., Dorival C., Simony M., Diallo A., Hezode C., De Ledinghen V., Larrey D., Haour G., Bronowicki J.P. (2019). Clinical Outcomes in Patients with Chronic Hepatitis C after Direct-Acting Antiviral Treatment: A Prospective Cohort Study. Lancet.

[11] Verna E.C., Morelli G., Terrault N.A., Lok A.S., Lim J.K., Di Bisceglie A.M., Zeuzem S., Landis C.S., Kwo P., Hassan M. (2020). DAA Therapy and Long-Term Hepatic Function in Advanced/Decompensated Cirrhosis: Real-World Experience from HCV-TARGET Cohort. J. Hepatol..

[12] Nagata H., Nakagawa M., Asahina Y., Sato A., Asano Y., Tsunoda T., Miyoshi M., Kaneko S., Otani S., Kawai-Kitahata F. (2017). Effect of Interferon-Based and -Free Therapy on Early Occurrence and Recurrence of Hepatocellular Carcinoma in Chronic Hepatitis C. J. Hepatol..

[13] Nahon P., Layese R., Bourcier V., Cagnot C., Marcellin P., Guyader D., Pol S., Larrey D., De Lédinghen V., Ouzan D. (2018). Incidence of Hepatocellular Carcinoma After Direct Antiviral Therapy for HCV in Patients with Cirrhosis Included in Surveillance Programs. Gastroenterology.

[14] Kondili L.A., Quaranta M.G., Cavalletto L., Calvaruso V., Ferrigno L., D’Ambrosio R., Simonelli I., Brancaccio G., Raimondo G., Brunetto M.R. (2023). Profiling the Risk of Hepatocellular Carcinoma after Long-Term HCV Eradication in Patients with Liver Cirrhosis in the PITER Cohort. Dig. Liver Dis..

[15] Ioannou G.N., Green P.K., Berry K. (2018). HCV Eradication Induced by Direct-Acting Antiviral Agents Reduces the Risk of Hepatocellular Carcinoma. J. Hepatol..

[16] Kanwal F., Kramer J., Asch S.M., Chayanupatkul M., Cao Y., El-Serag H.B. (2017). Risk of Hepatocellular Cancer in HCV Patients Treated with Direct-Acting Antiviral Agents. Gastroenterology.

[17] Cheung M.C.M., Walker A.J., Hudson B.E., Verma S., McLauchlan J., Mutimer D.J., Brown A., Gelson W.T.H., MacDonald D.C., Agarwal K. (2016). Outcomes after Successful Direct-Acting Antiviral Therapy for Patients with Chronic Hepatitis C and Decompensated Cirrhosis. J. Hepatol..

[18] Tanaka Y., Ogawa E., Huang C.F., Toyoda H., Jun D.W., Tseng C.H., Hsu Y.C., Enomoto M., Takahashi H., Furusyo N. (2020). HCC Risk Post-SVR with DAAs in East Asians: Findings from the REAL-C Cohort. Hepatol. Int..

[19] Calvaruso V., Cabibbo G., Cacciola I., Petta S., Madonia S., Bellia A., Tinè F., Distefano M., Licata A., Giannitrapani L. (2018). Incidence of Hepatocellular Carcinoma in Patients with HCV-Associated Cirrhosis Treated with Direct-Acting Antiviral Agents. Gastroenterology.

[20] Lazarus J.V., Picchio C.A., Colombo M. (2023). Hepatocellular Carcinoma Prevention in the Era of Hepatitis C Elimination. Int. J. Mol. Sci..

[21] Reig M., Mariño Z., Perelló C., Iñarrairaegui M., Ribeiro A., Lens S., Díaz A., Vilana R., Darnell A., Varela M. (2016). Unexpected High Rate of Early Tumor Recurrence in Patients with HCV-Related HCC Undergoing Interferon-Free Therapy. J. Hepatol..

[22] Conti F., Buonfiglioli F., Scuteri A., Crespi C., Bolondi L., Caraceni P., Foschi F.G., Lenzi M., Mazzella G., Verucchi G. (2016). Early Occurrence and Recurrence of Hepatocellular Carcinoma in HCV-Related Cirrhosis Treated with Direct-Acting Antivirals. J. Hepatol..

[23] Casotto V., Amidei C.B., Saia M., Gregori D., Zanetto A., Fedeli U., Russo F.P. (2024). Mortality Related to HCV and Other Chronic Liver Diseases in Veneto (Italy), 2008–2021: Changes in Trends and Age-period-cohort Effects. Liver Int..

[24] D’Ambrosio R., Degasperi E., Lampertico P. (2021). Predicting Hepatocellular Carcinoma Risk in Patients with Chronic HCV Infection and a Sustained Virological Response to Direct-Acting Antivirals. J. Hepatocell. Carcinoma.

[25] Castéra L., Vergniol J., Foucher J., Le Bail B., Chanteloup E., Haaser M., Darriet M., Couzigou P., De Lédinghen V. (2005). Prospective Comparison of Transient Elastography, Fibrotest, APRI, and Liver Biopsy for the Assessment of Fibrosis in Chronic Hepatitis C. Gastroenterology.

[26] Galle P.R., Forner A., Llovet J.M., Mazzaferro V., Piscaglia F., Raoul J.L., Schirmacher P., Vilgrain V. (2018). EASL Clinical Practice Guidelines: Management of Hepatocellular Carcinoma. J. Hepatol..

[27] ISTAT Statistiche ISTAT. http://dati.istat.it/index.aspx.

[28] AIFA AIFA. http://www.agenziafarmaco.gov.it/content/registri-farmaci-sottoposti-monitoraggio.

[29] Kondili L.A., Gaeta G.B., Brunetto M.R., Di Leo A., Iannone A., Santantonio T.A., Giammario A., Raimondo G., Filomia R., Coppola C. (2017). Incidence of DAA Failure and the Clinical Impact of Retreatment in Real-Life Patients Treated in the Advanced Stage of Liver Disease: Interim Evaluations from the PITER Network. PLoS ONE.

[30] Krassenburg L.A.P., Maan R., Ramji A., Manns M.P., Cornberg M., Wedemeyer H., de Knegt R.J., Hansen B.E., Janssen H.L.A., de Man R.A. (2021). Clinical Outcomes Following DAA Therapy in Patients with HCV-Related Cirrhosis Depend on Disease Severity. J. Hepatol..

[31] Romano A., Angeli P., Piovesan S., Noventa F., Anastassopoulos G., Chemello L., Cavalletto L., Gambato M., Russo F.P., Burra P. (2018). Newly Diagnosed Hepatocellular Carcinoma in Patients with Advanced Hepatitis C Treated with DAAs: A Prospective Population Study. J. Hepatol..

[32] Waziry R., Hajarizadeh B., Grebely J., Amin J., Law M., Danta M., George J., Dore G.J. (2017). Hepatocellular Carcinoma Risk Following Direct-Acting Antiviral HCV Therapy: A Systematic Review, Meta-Analyses, and Meta-Regression. J. Hepatol..

[33] Kardashian A., Serper M., Terrault N., Nephew L.D. (2023). Health Disparities in Chronic Liver Disease. Hepatology.

